# Fitness consequences of different migratory strategies in partially migratory populations: A multi‐taxa meta‐analysis

**DOI:** 10.1111/1365-2656.13155

**Published:** 2019-12-24

**Authors:** Claire Buchan, James J. Gilroy, Inês Catry, Aldina M. A. Franco

**Affiliations:** ^1^ School of Environmental Sciences University of East Anglia Norwich Norfolk UK; ^2^ Centro de Ecologia Aplicada ‘Prof. Baeta Neves’ and InBio – Rede de Investigação em Biodiversidade e Biologia Evolutiva Instituto Superior de Agronomia Universidade de Lisboa Lisboa Portugal

**Keywords:** behavioural dimorphism, climate change, evolution of migration, migratory strategy, movement ecology, partial migration

## Abstract

1. Partial migration—wherein migratory and non‐migratory individuals exist within the same population—represents a behavioural dimorphism; for it to persist over time, both strategies should yield equal individual fitness. This balance may be maintained through trade‐offs where migrants gain survival benefits by avoiding unfavourable conditions, while residents gain breeding benefits from early access to resources.
2. There has been little overarching quantitative analysis of the evidence for this fitness balance. As migrants—especially long‐distance migrants—may be particularly vulnerable to environmental change, it is possible that recent anthropogenic impacts could drive shifts in fitness balances within these populations.
3. We tested these predictions using a multi‐taxa meta‐analysis. Of 2,939 reviewed studies, 23 contained suitable information for meta‐analysis, yielding 129 effect sizes.
4. Of these, 73% (*n* = 94) reported higher resident fitness, 22% (*n* = 28) reported higher migrant fitness, and 5% (*n* = 7) reported equal fitness. Once weighted for precision, we found balanced fitness benefits across the entire dataset, but a consistently higher fitness of residents over migrants in birds and herpetofauna (the best‐sampled groups). Residency benefits were generally associated with survival, not breeding success, and increased with the number of years of data over which effect sizes were calculated, suggesting deviations from fitness parity are not due to sampling artefacts.
5. A pervasive survival benefit to residency documented in recent literature could indicate that increased exposure to threats associated with anthropogenic change faced by migrating individuals may be shifting the relative fitness balance between strategies.

## Graphical abstract

The authors provide the first quantitative analysis of relative fitness in partially migratory populations. Contrary to existing assumptions concerning the evolution and maintenance of migratory behaviours, they find evidence that residency confers greater fitness than migration for certain taxonomic groups and is associated with a benefit to survival, not breeding success.

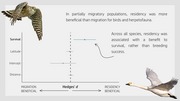

## Résumé

1. La migration partielle – dans laquelle les migrants et les résidents existent dans une seul population – représente un dimorphisme comportemental; pour qu'elles persiste au fil du temps, les deux stratégies doivent conférer la même valeur adaptive (fitness) individuelle. Cet équilibre peut être maintenu par un compromis où les migrants obtiennent des bénéfices de survie en évitant des conditions défavorables, tandis que les résidents obtiennent des avantages reproductifs grâce á un accès précoce aux ressources.
2. Peu d'analyses quantitatives ont été entreprise sur les preuves de cet équilibre de fitness. Étant donné que les migrants – en particulier les migrants de longue distance – peuvent être particulièrement vulnérables aux changements environnementaux, il est possible que les impacts anthropiques récents puissent entraîner une modification dans l’équilibre de fitness au sein de ces populations.
3. Nous avons testé ces prédictions en utilisant une méta‐analyse multi‐taxa. Sur 2,939 études examinées, 23 contenaient des informations appropriées pour la méta‐analyse, donnant 129 tailles d'effet.
4. Parmi eux, 73% (*n* = 94) ont déclaré un fitness supérieur pour les résidents, 22% (*n* = 28) un fitness supérieur pour les migrants, et 5% (*n* = 7) une égalité de fitness entre les deux. Après avoir pondéré les données par la précision, nous avons trouvé des avantages équilibrés sur le fitness sur l'ensemble des données, mais parmi les oiseaux et l'herpétofaune (les groupes les mieux échantillonnés), de manière cohérente, les résidents étaient associés à un meilleur fitness comparé à celle des migrants. Les bénéfices d'être résident étaient associés à la survie, et non au succès reproductif, et augmente avec le nombre d'années de données pendant lesquelles la taille d'effet a été calculé; cela suggérerait que les écarts par rapport à la parité de fitness ne sont pas dus à des artefacts d'échantillonnage.
5. Cet avantage de survie pour les résidents, documenté dans la littérature récente, pourrait indiquer qu'une exposition accrue aux menaces associées aux changements anthropiques rencontrés par les individus migrateurs pourrait modifier l'équilibre entre les stratégies.

## 1 INTRODUCTION

Migratory species are found across all major taxonomic groups (Dingle & Drake, 2007), an increasing number of which are recognized as partial migrants (Chapman, Brönmark, Nilsson, & Hansson, 2011b; Meller et al., 2016; Reid et al., 2018), wherein migratory and non‐migratory individuals exist within the same population of a species (Chapman, Brönmark, Nilsson, & Hansson, 2011a; Lundberg, 1988). Previously underrepresented in migration literature (Chapman et al., 2011a; Sekercioglu, 2010), partial migration has seen an increase in published studies only in recent years (Meller et al., 2016)—at least in part owing the greater empirical research enabled by advances in tracking technologies (Chapman et al., 2011a, 2011b; Reid et al., 2018). The emergence of rigorous study on this topic represents an opportunity to address unanswered questions surrounding the evolution and maintenance of partial migration (and behavioural polymorphisms in general), the ecological consequences of different migratory patterns and the evolution of migration itself (Chapman et al., 2011b; Sekercioglu, 2010).

Migratory behaviours typically arise where temporary spatial displacement is an advantageous response to environmental variation (Alerstam, Hedenström, & Åkesson, 2012; Dingle, 1980). The potential costs of migration are high: migratory individuals may encounter unfamiliar environments with novel threats, as well as the energetic costs of movement (Wikelski et al., 2003), predation risks (Lindström, 1989; Ydenberg, Butler, Lank, Smith, & Ireland, 2004) and temporal investment to the detriment of time otherwise invested in breeding fitness (Alerstam et al., 2012). The biological processes underlying the evolution of migration are little known (Griswold, Taylor, & Norris, 2010; Townsend, Frett, McGarvey, & Taff, 2018; Vélez‐Espino, McLaughlin, & Robillard, 2013), but in order to have evolved, migration must—in sufficient instances—offer a benefit relative to not migrating (‘residency’ hereafter) to either breeding success or survival (Griswold et al., 2010; Lundberg, 1988; McKinnon et al., 2010; Zúñiga et al., 2017).

Partial migration represents a behavioural dimorphism; in order for it to be maintained, either the two strategies yield equivalent fitness returns—an evolutionary stable state—or they confer overall balanced relative benefits which differ according to circumstance, known as a conditional strategy (Chapman et al., 2011b; Kokko, 2011; Lundberg, 1988). It follows, therefore, that in partially migratory populations residency may offer complementary fitness benefits to those offered by migration (Lundberg, 1988; Zúñiga et al., 2017). In the case of conditional strategies, these may refer to individual states such as sex or body condition (Hegemann, Marra, & Tieleman, 2015; Warkentin, James, & Oliphant, 1990), or external conditions, such as population density (Grayson & Wilbur, 2009) or environmental conditions (Chapman et al., 2011b; Lack, 1968; Lundberg, 1987; Meller et al., 2016). Additionally, the prevalence of each strategy within a population may itself influence the relative fitness benefits conferred by either (Kokko, 2011; Lundberg, 1987).

Two of the main demographic parameters controlling population size are breeding success and survival (Griswold, Taylor, & Norris, 2011; Lundberg, 1987)*,* though the extent of the influence of each parameter on population size may differ between populations (Morrison, Robinson, Clark, Risely, & Gill, 2013). Theories surrounding the maintenance of partial migration have hypothesized that the balance of benefits between migration and residency hinges on differential advantages to survival versus breeding success between the strategies (Griswold et al., 2010; Lundberg, 1988; Zúñiga et al., 2017). These generally predict that migration confers survival benefit as it allows individuals to escape unfavourable climatic conditions and low resource abundance, while residency promotes breeding success through early access to better resources—such as territories or breeding locations (Chapman et al., 2011b; Kokko, 2011; Lundberg, 1987). Although relative fitness benefits have been quantified in many partially migratory populations (Bai, Severinghaus, & Philippart, 2012; Hansen, Aanes, & Sæther, 2010; Hebblewhite & Merrill, 2011; Palacín, Alonso, Martín, & Alonso, 2017), the generality of this prediction across taxa has not been tested previously. Assessing the prevalence of fitness parity between migrants and residents—and any patterns evident in the deviation from this parity—has the potential to add to our understanding of the ontogeny of migratory behaviours, as well as shed light on how migratory species will respond to increasing anthropogenic threats.

Migratory individuals depend on a wide range of temporally and spatially distributed habitats and resources across the annual cycle, which is thought to expose migrants—especially long‐distance migrants—to increased potential risks (Both et al., 2010; Gilroy, Gill, Butchart, Jones, & Franco, 2016; Robinson et al., 2009; Wilcove & Wikelski, 2008). Rising temperatures have been linked to poleward range shifts in migratory species (Breed, Stichter, & Crone, 2013; La Sorte & Thompson, 2007), shorter migration distances (Heath, Steenhof, & Foster, 2012; Visser, Perdeck, Balen, & Both, 2009), earlier arrival times (Jonzén et al., 2006; Usui, Butchart, & Phillimore, 2017) and earlier breeding times (Both et al., 2004; Tomotani et al., 2017). Furthermore, the capacity of migratory species to adapt to climate change is not universal (Fraser et al., 2013; Robinson et al., 2009), and inability to do so has been linked to population declines (Møller, Rubolini, & Lehikoinen, 2008). Partial migration may confer some resilience to environmental change, since some individuals are not exposed to the threats posed by migration (Chapman et al., 2011b); indeed, partial migration has been shown to be a positive predictor of population trends in European birds (Gilroy et al., 2016). Climate change has been predicted to make residency increasingly beneficial and accordingly bring about a decrease in migratory tendency among partial migrants (Berthold, 2001; Pulido & Berthold, 2010). This may be particularly relevant in populations where selection pressures favouring migration are weaker, such as at lower latitudes, where the reduced seasonality—and associated lower variability in resources (Robinson et al., 2009; Somveille, Manica, Butchart, & Rodrigues, 2013)—means fitness benefits may be more closely balanced between resident and migrant strategies. Again, however, the generality of these patterns has not been tested across taxa.

The growing bank of research surrounding partial migration represents an unexplored opportunity for quantitative synthesis, rendered particularly timely by the growing impacts of global environmental change on migratory species (Robinson et al., 2009). Here, we employ a meta‐analytic approach to assess whether the individual fitness benefits of migration and residency are indeed balanced in partially migratory populations. We also evaluate the generality of patterns relating to the type of benefit—breeding success or survival—for either strategy. Additionally, we consider the potential influence of latitude and migratory distance on these relative benefits, further predicting that, were environmental change driving a change in balance, it would result in more benefits to residency in long‐distance migrants or low‐latitude systems.

## 2 MATERIALS AND METHODS

### 2.1 Data collection

We carried out a systematic search of studies published until December 2017 using the search terms outlined in Table 1 via ISI Web of Science and Google Scholar, without constraining our results to any specific taxonomic group(s). For each search phrase, we extracted all results that fell into any of the Web of Science‐defined categories deemed potentially relevant to partial migration (Behavioural Sciences, Biodiversity Conservation, Biology, Ecology, Entomology, Environmental Sciences, Environmental Studies, Evolutionary Biology, Fisheries, Marine Freshwater Biology, Ornithology, Zoology). For the results of the Google Scholar search, we extracted the first 120 results for each search phrase using a browser‐based web‐scraping tool (Data Miner, 2017). The search syntax differs slightly to that used for Web of Science; Google Scholar automatically inserts the Boolean operator ‘AND’ between all search terms unless another is specified. Furthermore, truncation is not recognized by Google, which instead uses automatic word stemming as part of a suite of ‘query expansion’ measures (Google, 2018).

**Table 1:** Search terms used to create unfiltered reference library

Database/Search engine	Search terms				
ISI Web of Science	benefits	AND	migration		
	benefits	AND	migration	AND	strategy
	benefits	AND	migratory	AND	strategy
	benefits	AND	partial	AND	migration
	benefits	AND	resident	AND	migrant
	consequences	AND	partial	AND	migration
	consequences	AND	partial	AND	strategy
	reproduct*	AND	benefits	AND	migration
	reproduct*	AND	partial	AND	migration
	fitness	AND	partial	AND	migration
	survival	AND	benefits	AND	migration
	survival	AND	partial	AND	migration
Google Scholar	benefits migration				
	benefits migration strategy				
	benefits migratory strategy				
	benefits partial migration				
	benefits resident migrant				
	consequences partial migration				
	consequences partial strategy				
	reproduction benefits migration				
	reproductive benefits migration				
	reproduction partial migration				
	reproductive partial migration				
	fitness partial migration				
	survival benefits migration				
	survival partial migration				

Asterisks in ISI WoS search terms indicate truncation, whereby words with multiple endings to the root word are included in the search.

Following Stewart and colleagues (Stewart, Pullin, & Coles, 2007; and cited elsewhere as good practice—Côté, Curtis, Rothstein, & Stewart, 2013, p. 47), we also conducted supplementary literature searches in order to add to—and validate the accuracy of—the results of the keyword search. These consisted of searching the reference lists of papers already in our accepted reference library and of the narrative review of partial migration by (Chapman et al., 2011b). We also carried out additional searches with altered keywords to ensure our results encompassed taxonomic groups whose literature employs different migration terminology (e.g. diadromy in fish).

We filtered the resulting papers according to their potential relevance to our research question. Filtering was done initially by abstract, then again by full‐text, retaining any studies for which it appeared possible they could fulfil the following criteria:

Does the study compare either a resident and migrant population of the same species or a short‐distance migrant and long‐distance migrant population of the same species?
Does the study measure outcomes deemed by its authors to be a potential consequence of migratory strategy?
Does the study measure outcomes deemed by its authors to be ecologically beneficial/detrimental to the survival or reproductive success of individuals?
Can these outcomes be considered direct indicators of fitness?
Does the study report extractable data necessary for calculation of effect measures?
Are the data reported either raw observations or predicted by models fitted to raw data? (i.e. experimental data and theoretical models excluded.)

We included studies comparing short‐distance migrants to long‐distance migrants (in addition to those comparing residents to migrants) in an attempt to encompass more of the spectrum of migratory differences, and acknowledging that distinctions between residents and migrants may in any case not necessarily be strictly dichotomous (Reid et al., 2018). We only considered effect sizes relating to traits we deemed directly indicative of survival or breeding success; this resulted in a smaller sample size by excluding measures of, for instance, oxidative stress, predation risk and body size (see Table S1), but ensured that metrics could be reliably interpreted as direct measures of fitness. See Data sources section for a list of all data sources used in the analysis.

### 2.2 Data extraction

We extracted means and standard deviations for all reported results that fulfilled the inclusion criteria. For each effect size, we also extracted sample size, year(s) over which the data were gathered, species, location of study, migratory distance and type of fitness metric (breeding success or survival). Means and standard deviations were derived from raw data where these were given, and otherwise were model‐predicted (from models fitted to raw data—see *Inclusion criteria*). In instances where standard deviations were missing, we calculated these from standard errors or confidence intervals; bounded data were logit‐transformed prior to these calculations. Where data were presented only in graphical format, we used digitizing software (webplotdigitizer version 4.1; Rohatgi, 2018) to extract these. Means, standard errors and sample sizes were then used to calculate Hedges’ *d* standardized mean difference as a measure of effect size (Box S1; Hedges, 1981; Hillebrand & Gurevitch, 2016) using the metafor package in r (Viechtbauer, 2010). We arbitrarily assigned effect sizes positive (>0) when resident individuals showed a fitness benefit, and negative (<0) when migrants showed a benefit. Benefits were considered as such according to the interpretations of the individual paper authors.

Various measures of biological fitness exist, with different metrics more relevant for certain taxonomic groups/ecological systems than others. Indices of fitness were classed as pertaining either to breeding success (e.g. clutch size, offspring survival) or to survival (absolute survival, growth rate, see Table S1).

### 2.3 Meta‐analysis

We obtained overall predicted mean effect sizes (*d*) and their associated within‐study variance (*ψ*) using meta‐analytic random‐effects models via maximum‐likelihood estimation, weighting effect sizes by their inverse variance (1/*ψ*), a metric of precision/statistical power. We considered the resulting mean effect sizes as significant if the 95% confidence intervals did not include zero. As individual papers frequently yielded multiple effect sizes, we included ‘study’ as a random effect to account for within‐study non‐independence (Mengersen, Jennions, & Schmid, 2013). Even within studies, the methods and systems associated with each effect size were not identical, so the individual identity (ID) of each effect size was also included as a random effect (Viechtbauer, 2010). We assessed the presence of heterogeneity using Cochran's *Q* test, a significant result of which indicates that variation between effect sizes is greater than the expected result of chance sampling variability (Viechtbauer, 2007). We created models for each taxonomic group individually (bird, fish, herpetofauna and mammal), as well as across all species.

### 2.4 Meta‐regression

To explore causes of heterogeneity and assess the influence of ecological predictors on the relative benefits of residency, we then added moderators (equivalent to fixed effects) to a meta‐analytic random‐effects model, with taxonomic group as an additional random effect. The response variable in these models was again the standardized effect size (*d*), representing study‐observed fitness benefit of residency over migration. We tested the influence of three moderators: latitude, migratory distance and type of fitness metric. Latitude was the approximate latitude of area shared by migrants and residents—that is the breeding ground if non‐breeding partial migrants and the wintering ground if breeding partial migrants. The distance moderator was the natural log (to achieve a normal distribution) of the one‐way distance residents ‘saved’ by not migrating. In cases where residents were truly resident (*n* = 109), this was simply equal to the distance travelled by migrants. In cases where ‘residents’ were in fact short‐distance migrants being contrasted with long‐distance migrants (*n* = 20), the ‘distance saved’ was the difference in distance travelled. Type of fitness metric was a two‐level categorical predictor based on whether the fitness measure related to survival or to breeding success (Table S1). Continuous moderators (latitude and distance) were scaled and centred prior to analysis.

We followed an information theoretic approach to assess the influence of moderators, in which we fitted random‐effects models with all possible combinations of the main effects. We also considered the potential influence of two‐way interactions, but found these to be unimportant and excluded these from further analysis. This resulted in a candidate set of eight models. We used Akaike's information criterion adjusted for small sample size (AICc) to compare model fit and used the glmulti package (Calcagno & de Mazancourt, 2010) to average over models in each candidate set within two AICc units of the best‐ranked model to obtain AICc‐weighted average coefficients and predictions (Burnham & Anderson, 2002). We examined the 95% confidence intervals of model‐averaged coefficients in order to assess the importance of moderators.

### 2.5 Study duration

We assessed the impact of study duration (number of years’ data contributing to effect size estimates) on the detection of fitness differences, to evaluate whether deviations from the expected parity of fitness between residents and migrants were more likely to arise in shorter studies (and hence potentially reflect sampling artefacts). We fitted a meta‐analytic random‐effects model to measures for all species, with study duration as a continuous moderator on standardized effect size, and inferred moderator significance from coefficient confidence intervals. Multi‐level meta‐analytical models carried out in metafor automatically conduct an omnibus test for the significance of the influence of parameters on effect size (Viechtbauer, 2010); we also considered the results of this when interpreting the results of the model.

### 2.6 Publication bias/sensitivity analysis

We evaluated the dataset for publication bias—which can result in unreliable conclusions (Jennions, Kahn, Kelly, & Kokko, 2013)—using a modification of Egger's regression test (Sterne & Egger, 2005). We fitted a multi‐level random‐effects model to the data with effect size standard deviation (√*ψ*) as a moderator; if the intercept of this model differs significantly from zero (*p* < .1), then the data are considered biased (Habeck & Schultz, 2015; Jennions et al., 2013). As meta‐analyses can be susceptible to the effects of outlying datapoints (Viechtbauer & Cheung, 2010), we assessed the sensitivity of our results. Following (Habeck & Schultz, 2015), we classified any effect size with a hat value (a measure of leverage: the influence of observed values on fitted values) of more than double the mean hat value of the dataset *and* standardized residuals greater than ±3 as an influential outlier (Stevens, 1984). Where such outliers existed, we reran the analyses without them to assess their influence on our results. Although a common approach in meta‐analyses, weighting by inverse variance has recently been argued to result in biased results in some instances (Hamman, Pappalardo, Bence, Peacor, & Osenberg, 2018). We therefore also ran all analyses weighting by sample size, but found no difference in our results. We therefore report results from the inverse variance weighted models in the remainder of the paper.

Unless stated otherwise, results given are model‐predicted standardized mean effect sizes (*d*) and associated 95% confidence intervals. All statistical analyses were carried out in r version 3.4.2 (R Core Development Team, 2018).

## 3 RESULTS

Of 2,939 studies found in the systematic literature search, 23 fulfilled all inclusion criteria and contained suitable information for meta‐analysis. We extracted 129 fitness measures from these 23 studies, representing data from 18 species spread over twelve orders. Data relating to species from the order Passeriformes (perching birds) accounted for 44% (*n* = 57) of all effect sizes extracted. The dataset encompassed studies from twelve different countries, of which all but one (the Republic of Seychelles, contributing five datapoints) were in the Northern Hemisphere. Years of data collection spanned 38 years (1976–2013), but there was a strong skew towards more recent studies, with 84% of effect sizes collected between 2000 and 2013 (Figures S1–S3). Of these effect sizes, 73% (*n* = 94) reported higher fitness in residents; 22% (*n* = 28) reported higher fitness in migrants and 5% (*n* = 7) as being equal.

### 3.1 Meta‐regression

**Figure 1:**
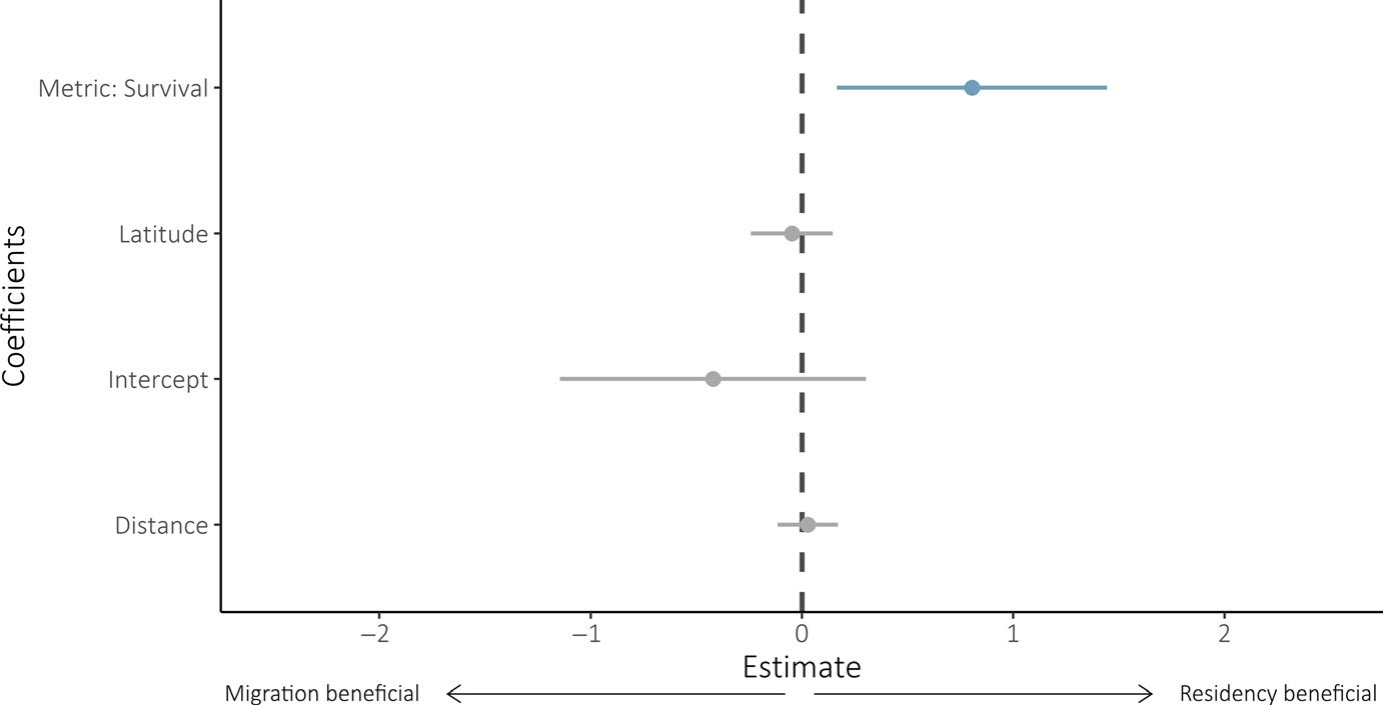
Model‐averaged coefficient estimates for fitness measures (*n* = 129). Positive estimates indicate a benefit to residency and negative values indicate a benefit to migration. Error bars represent 95% confidence intervals. Confidence intervals of blue points exclude zero, and those of grey points include zero

For meta‐regression models fitted to all measures (*n* = 129), model selection revealed metric type to be an important predictor of whether either migratory strategy was advantageous, with residency yielding benefits for survival but not breeding success metrics (model‐averaged coefficient estimate: 0.81, CIs: 0.17, 1.44; Tables 2 and 3; Figure 1). Neither latitude nor migratory distance emerged as important predictors of strategy benefits (model‐averaged coefficient estimates—latitude: −0.05, CIs: −0.24, 0.15, distance: 0.03, CIs: −0.12, 0.17; Table S2).

**Table 2:** Model‐averaged coefficients from models fitted to dataset of effect sizes (*n* = 129) within two AICc units of the top model (*n* = 3) showing influence of moderator variables on standardized effect size

Moderator	Estimate	Unconditional variance	No. models	Importance	L95%	U95%
Distance	0.028	0.005	1	0.207	−0.115	0.171
Latitude	−0.047	0.01	1	0.26	−0.241	0.146
Intercept	−0.421	0.136	3	1	−1.145	0.303
**Metric: survival**	**0.805**	**0.106**	**3**	**1**	**0.165**	**1.444**

Bold indicates important predictors, as determined from 95% confidence intervals.

**Table 3:** Candidate models fitted to dataset of effect sizes (*n* = 129) ranked by Akaike's information criterion adjusted for small sample size (AICc)

Model	AICc	Delta AICc	Weights
d ~ 1 + Metric type	401.8037	0	0.383356
d ~ 1 + Metric type + Latitude	403.2428	1.4391	0.186686
d ~ 1 + Metric type + Distance	403.6943	1.8906	0.14896
d ~ 1 + Metric type + Latitude + Distance	404.109	2.3053	0.121066
d ~ 1	405.4768	3.6731	0.061096
d ~ 1 + Latitude + Distance	406.5588	4.7551	0.035568
d ~ 1 + Distance	406.5911	4.7874	0.034997
d ~ 1 + Latitude	407.018	5.2143	0.028271

### 3.2 Individual taxonomic group models

**Figure 2:**
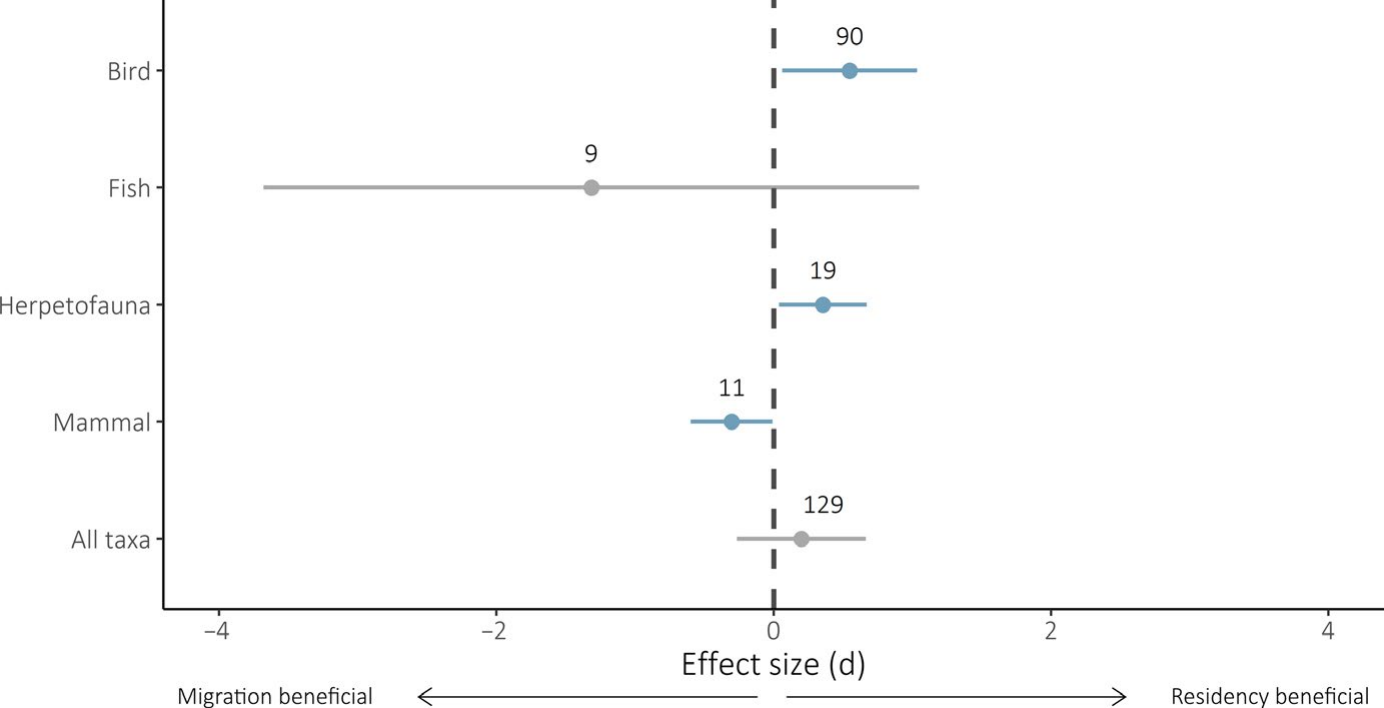
Effect sizes (*d*) predicted by individual meta‐analytic random‐effects models fitted to taxonomic subsets of all fitness measures (*n* = 129). Effect sizes greater than zero (dashed no‐effect line) indicate a benefit to residency, and effect size values below zero indicate a benefit to migration. Error bars represent 95% confidence intervals. Confidence intervals of blue points exclude zero, and those of grey points include zero

Across all fitness measures for all species (*n* = 129), we found no significant difference in fitness for migrants or residents (*d* = 0.20, CIs: −0.27, 0.66; Figure 2). However, there were differences within taxonomic groups: residency conferred fitness benefits for birds (*d* = 0.55, CIs: 0.06, 1.03) and herpetofauna (*d* = 0.35, CIs: 0.04, 0.67), while migration was beneficial to mammals (*d* = −0.30, CIs: −0.60, −0.01), and neither strategy conferred a fitness benefit to fish (*d* = −1.31, CIs: −3.68, 1.05). For all taxonomic groups barring mammals, Cochran's *Q* test was significant, indicating substantial unexplained heterogeneity among effect sizes (Table S3).

### 3.3 Study duration and publication bias

**Figure 3:**
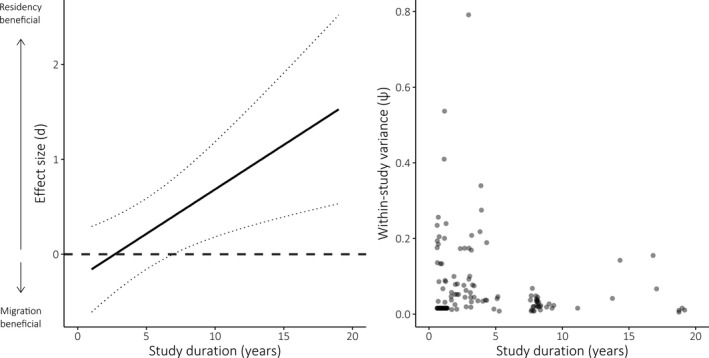
Left: Predicted effect of study duration on effect size (*d*) for fitness measures of all species (*n* = 129). Positive effect size values indicate a benefit to residency, and negative values indicate a benefit to migration. Dotted lines indicate 95% confidence intervals. Right: Raw values of effect size variance varying with study duration

Mean benefits of residency over migration increased with the number of years over which effect sizes were calculated (coefficient estimate: 0.09, CIs: 0.02, 0.28, QM *p*‐value: .0049; Figure 3). Among models that found a significant effect of migratory strategy on fitness, only the herpetofauna subset showed any evidence of publication bias (intercept *p*‐value: .0113; Table S4). This was, however, the group with the fewest studies contributing data, and Egger's test is potentially unreliable in cases with few studies (Cochrane Collaboration, 2011). Sensitivity analysis did not reveal any influential outliers in the dataset (Figure S4).

## 4 DISCUSSION

Little is known about the fitness balances of migratory strategies necessary for the evolutionary maintenance of partial migration, or the extent to which global environmental change may be altering this balance through differential impacts on migratory individuals. We provide evidence that many partially migratory populations studied in recent decades show greater fitness in resident individuals, with these benefits generally relating to survival rather than breeding success. These results are contrary to predictions surrounding the ontogeny of migratory behaviours (Chapman et al., 2011b; Lundberg, 1987), but are in line with predictions relating to the impacts of recent anthropogenic change on the survival of migratory individuals (Berthold, 2001). The presence of residual heterogeneity in all models indicates that additional unexplored environmental factors may also be influencing effect sizes.

### 4.1 Survival benefits of residency

Seasonal variability is considered one of the main drivers of migration, where migration may have evolved as a means of enhancing survival by allowing individuals to escape unfavourable conditions (Lundberg, 1987). This meta‐analysis provides evidence that residency, rather than migration, confers a survival benefit—a result obtained from a synthesis of data gathered over the last four decades, a time marked by the cumulative impacts of increasing anthropogenic environmental change (IPCC, 2013). Changes in seasonality—particularly warmer winters in the Northern Hemisphere (IPCC, 2013)—could plausibly alter the fitness costs associated with enduring a (formerly) harsh winter or undertaking migration (Berthold, 2001, 2003). Milder winters (Nilsson, Lindström, Jonzén, Nilsson, & Karlsson, 2006) and year‐round availability of artificial food sources (see Satterfield, Marra, Sillett, & Altizer, 2018) may render it unnecessary to undergo the costs of migration to escape unfavourable conditions, while advancing spring temperatures also favour residents, as they are less likely than migrants to suffer phenological mismatches (Pulido & Berthold, 2010). By forgoing migration, residents are better able to exploit earlier optimal conditions, on which migrants may miss out if unable to advance sufficiently their spring arrival (Møller et al., 2008). Residents are also in a better position than migrants to react to environmental cues on the breeding grounds (Cobben & van Noordwijk, 2017; Visser, Both, & Lambrechts, 2004). Simultaneously, anthropogenic activity may be making migratory journeys increasingly hazardous. Migratory individuals’ exposure to and reliance on a greater range of resources and geographic regions puts them at greater risk to the dangers of an increasingly unpredictable world (Gilroy et al., 2016; Vickery et al., 2014). The predicted increase in extreme weather events brought about by climate warming—notably droughts at low latitudes—may be particularly detrimental to migratory species (IPCC, 2013; Robinson et al., 2009). Increasing infrastructure and land‐use change may also add to mortality risks associated with migration. The construction of power lines, for instance, is associated with greater mortality in migrating birds (Palacín et al., 2017), while agricultural intensification, damming and hunting are all thought to have negative consequences for migratory birds (Adams, Small, & Vickery, 2014; Vickery et al., 2014).

Various other mechanisms could also explain the observed survival benefit of residency over migration. For instance, higher rates of emigration among migrants compared to residents could artificially increase ‘apparent survival’ in residents, such that our observed results reflect sampling error. However, as migrants tend to show high philopatry (Newton, 2008), it seems unlikely that this would be the sole driver of our results. Alternatively, as discussed above, the observed survival benefits of residency could reflect other individual traits such as sex, body size, and age, if these traits are themselves linked to migratory strategy (Chapman et al., 2011b). However, for this to explain a pervasive survival benefit of residency across studies, the underlying trait linkages would have to be common across species, which seems unlikely. A further possibility is that parity of fitness is not in fact required for partial migration to persist over evolutionary time. It is possible for some behavioural polymorphisms to be maintained despite differences in mean fitness, if there is a high variability associated with the more beneficial strategy (Calsbeek, Alonzo, Zamudio, & Sinervo, 2002). If, within a population, residency offers on average a greater survival benefit, but is a high‐risk strategy associated with a large variance in survival, a migratory strategy could also persist within the population despite lower mean fitness. Nevertheless, a number of studies have reported that residency is increasing in certain species (Hebblewhite & Merrill, 2011; Meller et al., 2016; Van Vliet, Musters, & Ter Keurs, 2009), and migration distances declining (Berthold, 2001; Meller et al., 2016; Visser et al., 2009)—findings which lend credence to an association between differential strategy fitness and recent anthropogenic change. Given the widespread incidence of partial migration across ecosystems, it is likely that responses to climate changes will be far from uniform across species (Chapman et al., 2011b; Griswold et al., 2011), and not necessarily straightforward (Nilsson et al., 2006).

We did not find a benefit to breeding success of residency, contrary to expectations based on their presumed greater capacity to respond to phenological mismatches and achieve early access to breeding resources (Pulido & Berthold, 2010). Theoretical models indicate that, at least for populations that share a breeding range, improved wintering conditions in the breeding range can result in better productivity for both migrants and residents, in addition to improved survival for residents (Griswold et al., 2011). If this were the case, we would not expect to detect breeding measures having an influence on the relative benefits of migratory strategies, as these would be balanced. Rather, this would simply contribute to a survival benefit of residency.

### 4.2 Latitude and migratory distance

Although the direction of the model‐averaged coefficient estimates for latitude and migratory distance were in line with our predictions (that residency should be increasingly beneficial in long‐distance and low‐latitude systems), both were close to zero and neither were statistically important (Figure 1; Table 1), indicating a high degree of uncertainty in these trends. The lack of a strong signal for the influence of migratory distance on the fitness returns of residency may be related to our controlling for taxonomic group. General between‐taxa differences in locomotive efficiency, body size and fluid dynamics (Alerstam et al., 2012; Alexander, 2002) mean different migratory distances are differentially adaptive between—and accordingly correlated with—different taxonomic groups. For the fitness measures included in our meta‐regression, mean (±*SD*) migratory distance for birds was 978.11 km (±1915.53), while for fish, herpetofauna and mammals was 17.77 km (±19.1), 0.69 km (±0.81) and 38.22 km (±4.38), respectively. The lack of distance effect may also indicate that the apparent survival benefit to residency is driven by increasingly mild wintering conditions experienced by residents, rather than by greater mortality risks associated with migration.

We predicted that the lesser seasonality associated with low latitudes would lead to lower selection pressures on migration, and therefore a more delicate balance between strategies, more likely to shift in response to environmental change. However, higher latitudes are currently seeing a greater impact of climate change (IPCC, 2013), leading to the opposing pressures of traditionally higher seasonality alongside a greater decrease in seasonality brought about by climate change—the individual effects of which it is not possible to tease apart here.

### 4.3 Taxonomic differences

Our results suggest the within‐taxonomic group variability in our data is less marked than the between‐group differences; in addition to the stark differences in migratory distance between taxonomic groups, between‐taxa variances in body size, general physiology and life histories may also be driving differences in relative fitness benefits and susceptibility to the effects of climate change. Altitudinal migrants, such as in the ungulate populations which comprised our mammal data, may benefit from climate change‐induced longer vegetation growth periods, resulting in comparatively more forage of higher nutritional value in the higher‐altitude migrant ranges (Rolandsen et al., 2017). Differences between taxa may also not necessarily be down to direct taxonomic differences; we did not, for instance, distinguish between different models of partial migration, which differ according to which season (breeding or non‐breeding) residents and migrants are allopatric (Chapman et al., 2011b). These different models may result in different benefits to either strategy. A reduction in resource variability at a shared non‐breeding range is predicted to improve resident breeding success, while the same for a shared breeding range should bring about higher survival in residents (Griswold et al., 2011). Non‐breeding partial migration was much more common in our data for birds, fish and herpetofauna, while all mammal fitness measures were from breeding partial migrants. Additionally, differences between the highly variable migratory systems found in fish—freshwater/marine/estuarine, catadromous/anadromous—may go some way towards explaining variance within that group. Indeed, there is an argument to move away from traditional dichotomous models of partial migration in general, which—while useful—may ultimately be more simplistic than realistic (Reid et al., 2018).

### 4.4 Study duration

That we found residency to be increasingly beneficial as individual study duration suggests that deviations from parity in fitness benefits detected in our meta‐analyses were unlikely to be due to sampling artefacts. Furthermore, if individual fitness benefits were balanced between strategies through facultative migratory tendency—with individuals switching strategy between years—we would expect longer‐running studies to be more likely to find parity between strategies, but we find the opposite result. This also implies that short‐term studies may be inadequate as a means of uncovering differences in demographic parameters between migratory strategies. Similar results have been found by Pearce‐Higgins and colleagues (Pearce‐Higgins et al., 2015), whose recommendations concerning the importance of long‐term studies as a means of determining impacts of climate change we echo.

### 4.5 Future recommendations

This study represents a step towards a more comprehensive understanding of migratory strategies within partial migrants. The results of this meta‐analysis are in part a reflection of the nature of the available literature the concerning partial migration. Taxonomic biases, particularly the ornithocentrism in animal migration literature found elsewhere (Bauer et al., 2009), are partly a result of migratory behaviour being more common in certain groups and partly a reflection of feasibility: species more readily tracked and monitored are more likely to be the subject of studies relevant to this topic. Similarly, while the prevalence in this study of data from the Northern Hemisphere is in part a product of a more general bias found across ecological literature (Amano & Sutherland, 2013; Martin, Blossey, & Ellis, 2012), there is also greater prevalence of terrestrial migratory species in the Northern Hemisphere due to a combination of high seasonal variability and greater land mass (Somveille, Rodrigues, & Manica, 2015).

That we had a strong temporal skew towards more recent years (Figure S3) is unsurprising; as well as the increase in ecological research over time (Hillebrand & Gurevitch, 2016), partial migration as a topic has become more prominent in recent years, and rendered more feasible as tracking methodologies become more advanced. The study is subject to certain practicalities of meta‐analyses—such as the necessary exclusion of studies not reporting the required statistics for calculation of our chosen standardized effect size. Statistical rigour and quality of reporting have improved with time (Hillebrand & Gurevitch, 2016)—making recent papers more suitable for inclusion in meta‐analyses. The continuation of these trends may better enable future temporal analyses of relative fitness benefits, which may shed more light on responses to increasing anthropogenic influence.

## 5 CONCLUSIONS

We provide evidence that residency results in higher fitness than migration in certain partially migratory populations and that residency confers a greater benefit to survival than to breeding success. While not conclusive, this accords with the prediction that global environmental change may be altering the fitness balance in favour of residency (Berthold, 2001), through milder climatic conditions lessening pressures to migrate, and increased mortality risks associated with migration. If accurate, this indicates that anthropogenic change may alter selection pressures to increasingly promote residency—or, indeed, promote plasticity in migratory strategy in response to environmental unpredictability (Reid et al., 2018). Despite the growing literature devoted to partial migration, only twenty‐three studies were ultimately suitable for inclusion in this meta‐analysis. Continued research, especially examining direct fitness measures, coupled with improved/more standardized reporting (sample sizes, measures of variance), will facilitate deeper investigation into the topic, while our results concerning study duration point to the value of long‐term studies. Climate warming is predicted to continue at an unprecedented rate, with significant implications for global biodiversity (IPCC, 2013; Parmesan, 2006). Understanding whether migratory species may be able to mediate its negative consequences—and the demographic processes through which this may occur—is critical for effective conservation measures (Newson et al., 2009), while also providing an opportunity to shed light on the evolution of migratory behaviours.

## AUTHORS' CONTRIBUTIONS

C.B., J.J.G. and A.M.A.F. designed the study. C.B. collected the data, conducted the statistical analyses and wrote the manuscript. A.M.A.F. and J.J.G. provided statistical advice. A.M.A.F., I.C. and J.J.G. critically revised the manuscript. All authors (C.B., J.J.G., I.C. and A.M.A.F.) contributed to interpreting results and gave final approval for publication.

## Data Availability

The dataset underlying the analyses described in this study is available from the NERC Environmental Information Data Centre: https://doi.org/10.5285/1a4e8d59-e112-4de6-a06b-9ea47ff15815 (Buchan, Gilroy, Catry, & Franco, 2019). Relevant r code is included as part of the Supporting Information.
