# Supplementary material for: Fitness consequences of different migratory strategies in partially migratory populations: A multi‐taxa meta‐analysis
Source: J Anim Ecol. 2019 Dec 24;89(3):678–90. doi: 10.1111/1365-2656.13155 (PMC7078763; doi:10.1111/1365-2656.13155)
Supplement: Supplementary file 1 [file JANE-89-678-s001.pdf]

Table S1. Breakdown of direct fitness measures encountered classified according to survival or breeding success. We classified metrics as directly or indirectly indicative of fitness, calculating effect sizes only for measures considered direct. Survival metrics excluded for being too indirect: body condition, body fat, body height, body mass, body size, food availability, growth efficiency, gut fullness, haematocrit, immune system function, and oxidative stress. Breeding metrics excluded for being too indirect: egg size, offspring size, breeding phenology

Fitness measure	Metric-type	n Effect sizes calculated
Absolute survival	Survival	66
Breeding lifespan	Survival	1
Individual growth rate	Survival	8
Breeding attempts	Breeding	5
Breeding frequency	Breeding	1
Clutch frequency	Breeding	3
Clutch size	Breeding	9
Surviving offspring	Breeding	23
Success	Breeding	13

Box S1: Hedges' *d*:

$$d = \frac{(\overline{x}_T - \overline{x}_C)}{S} \times J$$

Where S represents pooled standard deviation:

$$S = \sqrt{\frac{(n_T - 1){SD_T}^2 + (n_C - 1){SD_C}^2}{n_T + n_C - 2}}$$

And J represents a bias correction factor:

$$J = \frac{\Gamma \frac{n_T + n_C - 2}{2}}{\Gamma \sqrt{\frac{n_T + n_C - 2}{2}} \; \frac{n_T + n_C - 3}{2}}$$

$\overline{x}_T$ – mean treatment response

$\overline{x}_C$ – mean control response

n_T – treatment sample size

n_C – control sample size

SD_T – standard deviation about treatment mean

SD_C – standard deviation about control mean

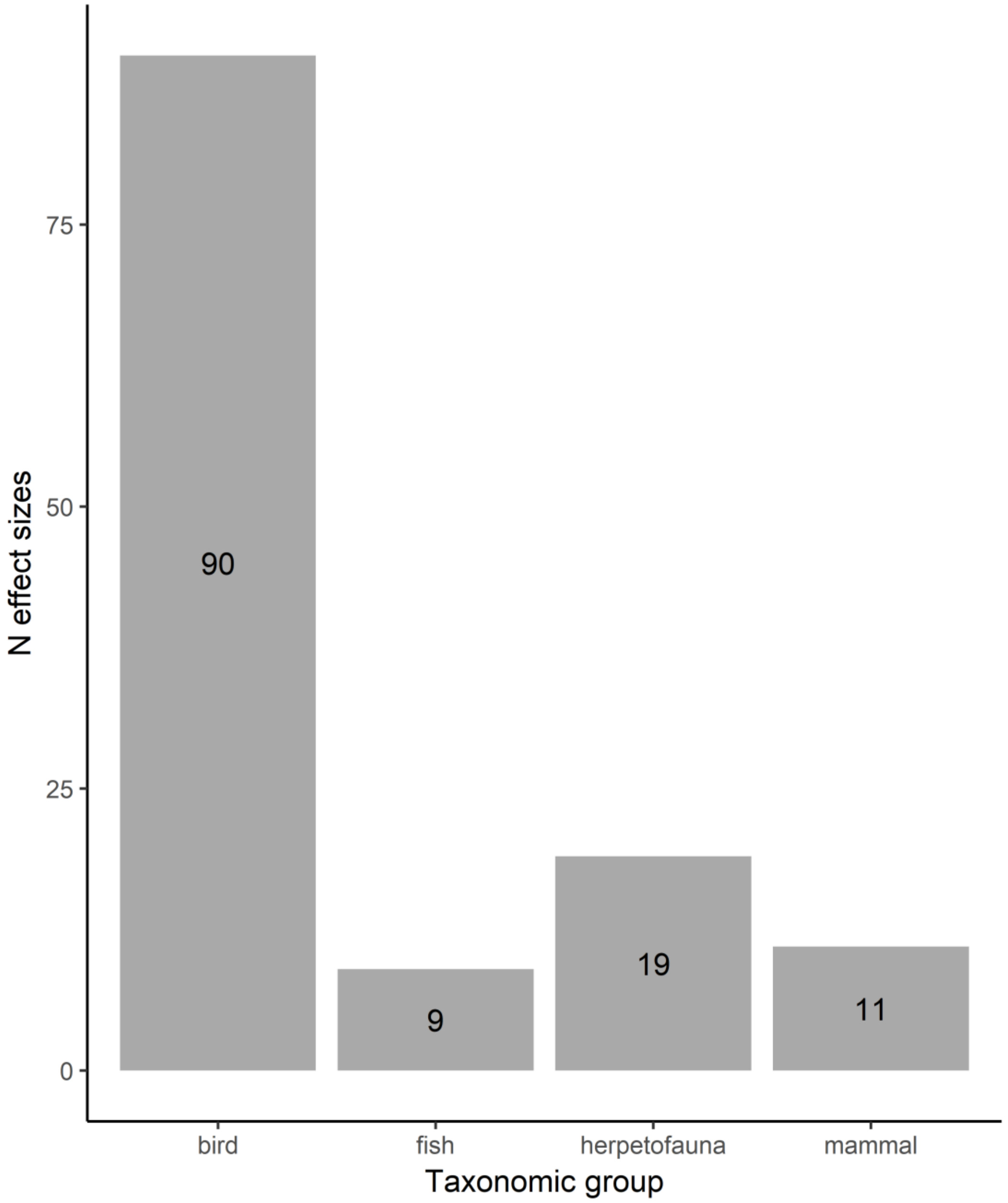

Figure S1. Breakdown of effect sizes by taxonomic group.

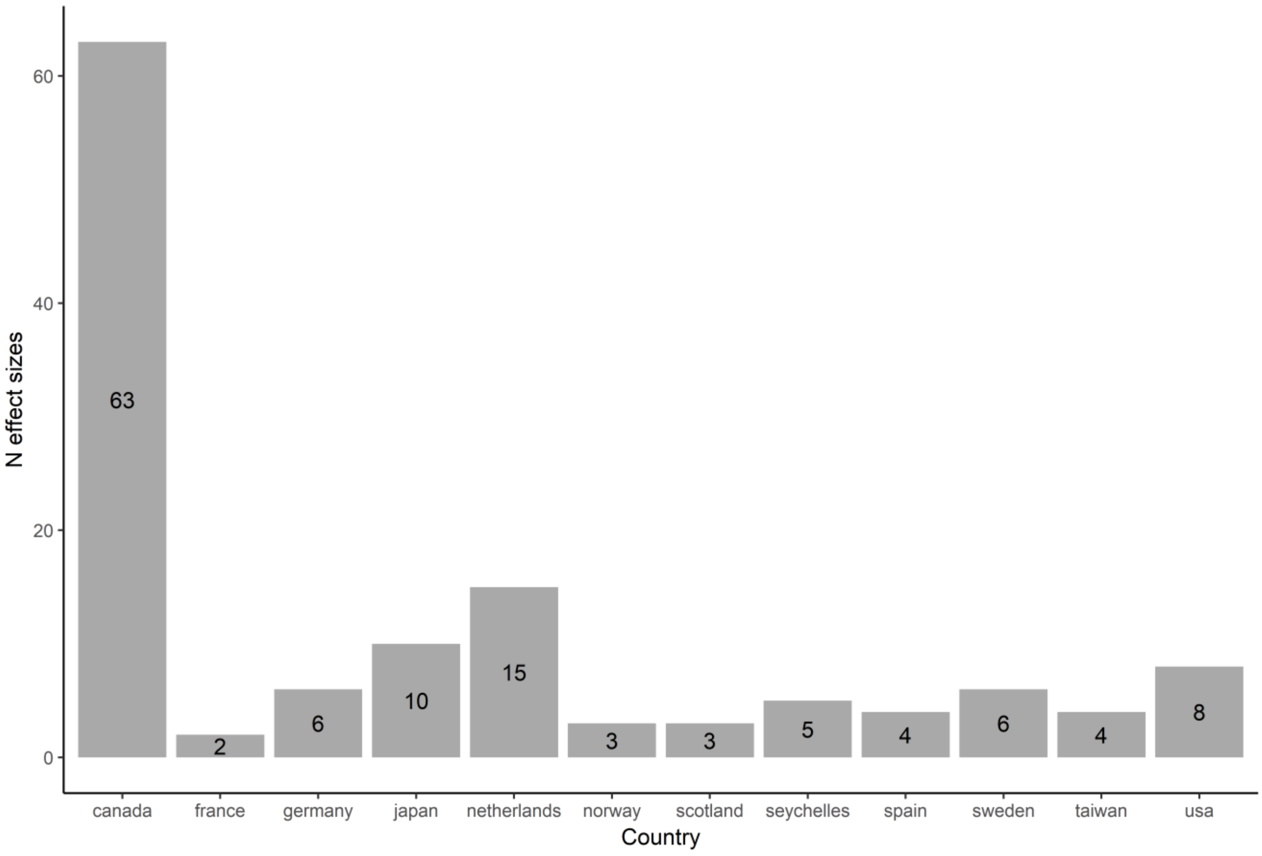

Figure S2. Breakdown of effect sizes by country in which study took place.

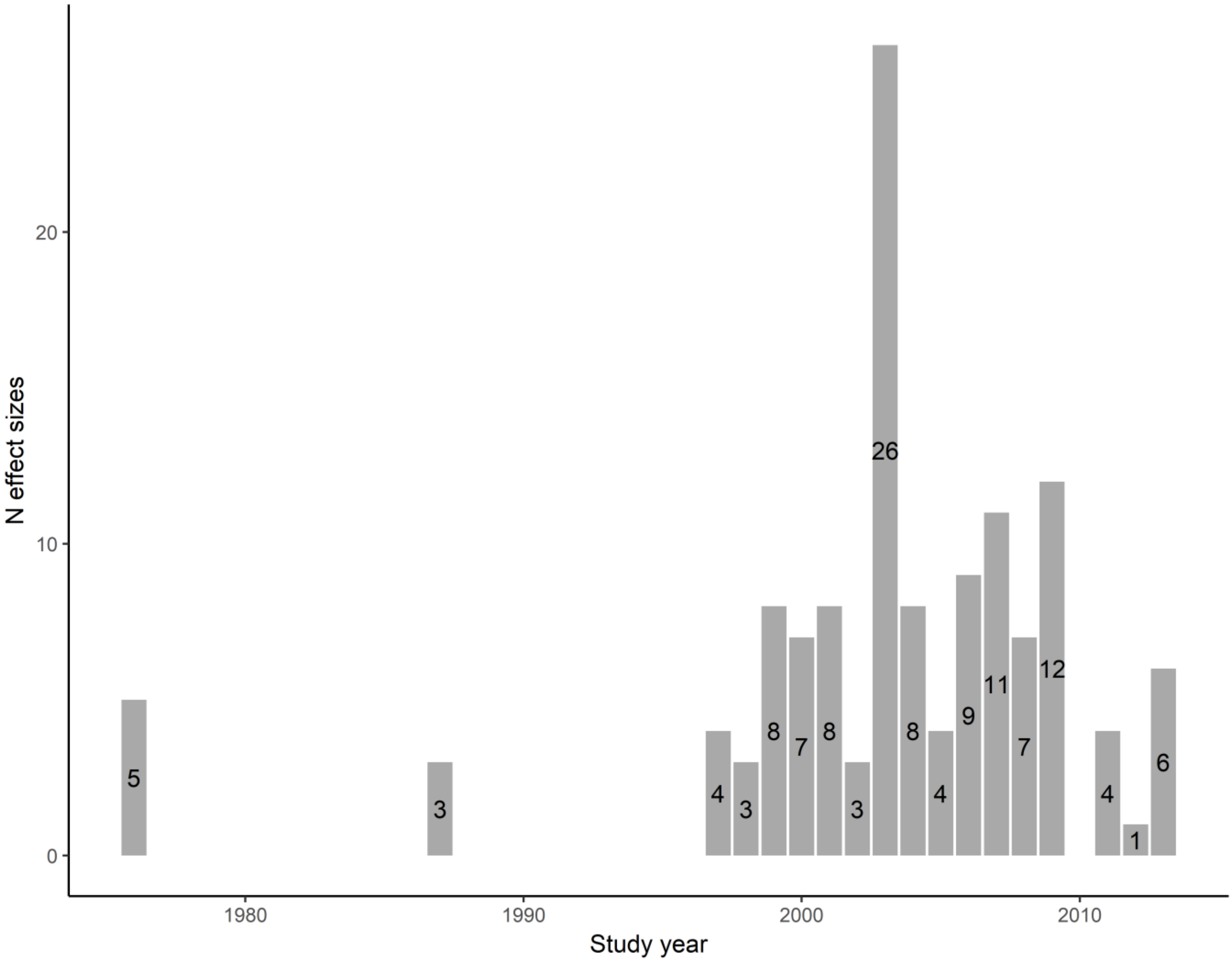

Figure S3. Breakdown of effect sizes by year in which data were collected. Median year of data collection is used for effect sizes calculated over multiple years.

Table S2. Coefficient estimates and model statistics for meta-regression models over which averaged coefficients were obtained. All models included effect size identity nested within study identity and taxonomic group as random effects.

	Coefficient estimates	[95% CIs]	p-value							Model statistics			
Model rank	Metric: survival			Latitude			Distance			Heterogeneity	Omnibus moderator test	AICc	Weight
1	0.8102	[0.1719, 1.4486]	0.0129	-	-	-	-	-	-	QE = 5207.0049<br>p < .0001	QM = 6.1891<br>p = 0.0129	401.8037	0.38336
2	0.8148	[0.1813, 1.4483]	0.0117	-0.1816	[-0.5856, 0.2225]	*0.3784*	-	-	-	QE = 5206.7424<br>p < .0001	QM = 7.0453<br>p = 0.0295	403.2428	0.18669
3	0.7776	[0.1307, 1.4244]	0.0185	-	-	-	0.1344	[-0.3003, 0.5691]	0.5446	QE = 4315.5184<br>p < .0001	QM = 6.4453<br>p = 0.0398	403.6943	0.14896

Table S3. Results of individual meta-analytic models estimating mean effect size. Negative mean effect sizes indicate a possible benefit of migration; positive mean effect sizes indicate a possible benefit of residency. Models with significant mean effect sizes are highlighted in bold. All models included effect size identity nested within study identity as random effects.

Dataset	N effect sizes	Estimated mean effect size	Lower 95% CI	Upper 95% CI	*P-value*	Heterogeneity statistics
All	129	0.1996	-0.2656	0.6647	*0.4004*	Q = 5405.0940<br>p < .0001
**Birds**	**90**	**0.5475**	**0.0622**	**1.0328**	***0.0279***	Q = 3799.1209<br>p < .0001
Fish	9	-1.3148	-3.6792	1.0495	*0.2757*	Q = 835.1767<br>p < .0001
**Herpetofauna**	**19**	**0.3545**	**0.0377**	**0.6713**	***0.0283***	Q = 125.9647<br>p < .0001
**Mammals**	**11**	**-0.3041**	**-0.5986**	**-0.0095**	***0.0430***	Q = 59.3797<br>p < .0001

Table S4. Model results for Egger's regression test for publication bias on the main dataset and taxonomic subsets thereof. Significant effects (P < 0.10) are highlighted in bold.

Dataset	n Effect sizes	Intercept	Lower 95% CI	Upper 95% CI	*P-value*
**All**	**129**	**0.6803**	**0.0328**	**1.3278**	***0.0395***
Birds	90	0.1950	-0.4459	0.8360	*0.5509*
**Fish**	**9**	**4.6607**	**-0.4159**	**9.7374**	***0.0720***
**Herpetofauna**	**19**	**0.9299**	**0.2102**	**1.6496**	***0.0113***
Mammals	11	-0.1798	-1.2940	0.9343	*0.7517*

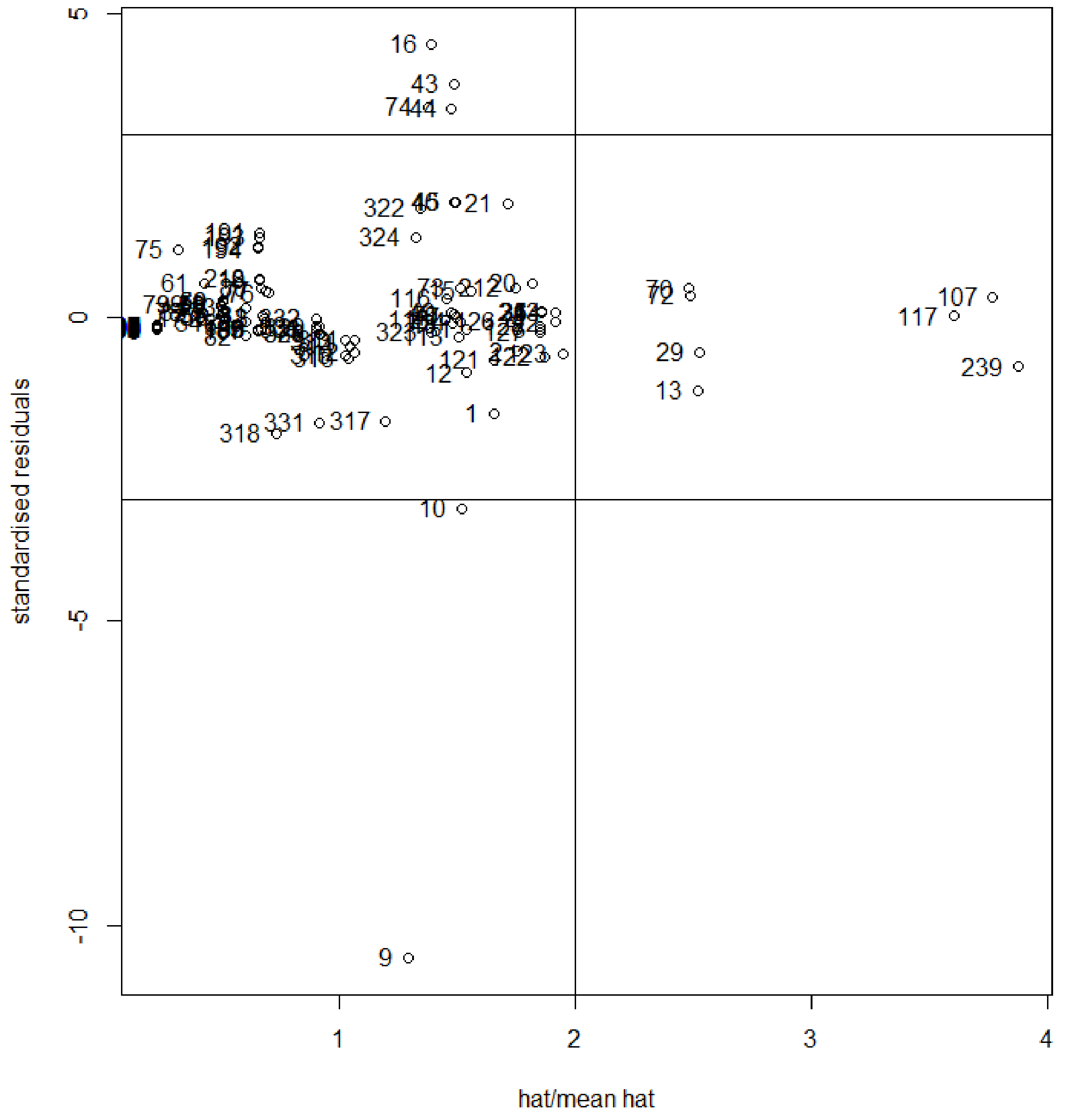

Figure S4. Sensitivity analysis plot for all effect sizes (n=129). No points were considered influential outliers – these would fall in the top or bottom right hand sections of the plot, indicating an effect size with both a hat value of more than twice the mean hat value (vertical line) and a standardised residual of more than ±3 (horizontal lines).

R CODE

```
data<-read.csv("DATASET_meta.csv", header=T)
names(data)
library(metafor)
library(glmulti)
#### meta-analysis ####
dat<-data
logdist<-log(dat$dist.saved)
sclogdist<-scale(logdist)
sclatitude<-scale(dat$latitude.dd)
dat<-cbind(dat,sclogdist,sclatitude)
ef<- escalc(measure="SMD", m1i=r.sdm.mean, sd1i=r.sdm.sd,
            n1i=n.res.sdm, m2i=ldm.mean, sd2i=ldm.sd, n2i=n.mig,
            data=dat, append=TRUE) #calculating effect size (yi) and variance (vi)
basicdir<-rma.mv(yi,vi, random = ~ 1 | study/ID, data=ef, method="ML")
summary(basicdir) #no effect of migratory strategy on measures
eggtest<-rma.mv(yi,vi, mod=~sqrt(vi), random = ~ 1 | study/ID, data=ef,
method="ML") #egger's regression test
summary(eggtest)  #testing publication bias
rstn<-rstandard(basicdir)
hat<-hatvalues(basicdir)/mean(hatvalues(basicdir))
plot(hat,rstn$resid,
     xlab="hat/mean hat",
     ylab="standardised residuals")
abline(h=-3)
abline(h=3)
abline(v=2)
text(hat,rstn$resid, labels=ef$ID, cex=1, pos=2) #no influential outliers (points
in top right or bottom right boxes)
#### meta-analysis - individually split by tax ####
cbird<-rma.mv(yi,vi,random = ~1 | study/ID, data=ef, subset=taxogroup.new=="bird",
method="ML")
summary(cbird)
cfish<-rma.mv(yi,vi,random = ~1 | study/ID, data=ef, subset=taxogroup.new=="fish",
method="ML")
summary(cfish)
cherp<-rma.mv(yi,vi,random = ~1 | study/ID, data=ef,
subset=taxogroup.new=="herpetofauna", method="ML")
summary(cherp)
cmam<-rma.mv(yi,vi,random = ~1 | study/ID, data=ef, subset=taxogroup.new=="mammal",
method="ML")
summary(cmam)
##### individual taxonomic pub bias tests ####
birdegg<-rma.mv(yi,vi, mod=~sqrt(vi), random = ~ 1 | study/ID,
data=ef,subset=taxogroup.new=="bird", method="ML")
summary(birdegg)
fishegg<-rma.mv(yi,vi, mod=~sqrt(vi), random = ~ 1 | study/ID,
data=ef,subset=taxogroup.new=="fish", method="ML")
summary(fishegg)
herpegg<-rma.mv(yi,vi, mod=~sqrt(vi), random = ~ 1 | study/ID,
data=ef,subset=taxogroup.new=="herpetofauna", method="ML")
summary(herpegg)
monotreme<-rma.mv(yi,vi, mod=~sqrt(vi), random = ~ 1 | study/ID,
data=ef,subset=taxogroup.new=="mammal", method="ML")
summary(monotreme)
#### meta-regression ####
dat<-subset(data, dist.saved!="NA")
logdist<-log(dat$dist.saved)
sclogdist<-scale(logdist)
sclatitude<-scale(dat$latitude.dd)
dat<-cbind(dat,logdist,sclogdist,sclatitude)
Cef<- escalc(measure="SMD", m1i=r.sdm.mean, sd1i=r.sdm.sd,
```

```
             n1i=n.res.sdm, m2i=ldm.mean, sd2i=ldm.sd, n2i=n.mig,
             data=dat, append=TRUE)
rma.glmulti.ran <- function (formula, data, ...) {
  rma.mv(formula, vi, random= ~1 | taxogroup.new/study/ID, data = Cef, method="ML",
...)
}
res<-glmulti(yi~benefit+sclatitude+sclogdist, data=Cef, level=1,
fitfunction=rma.glmulti.ran, crit="aicc", marginality = TRUE)
print(res)
tmp<-weightable(res)
tmp
"methods/html/setOldClass.html">setOldClass("rma.mv")
"methods/html/setMethod.html">setMethod('getfit', 'rma.mv', function(object, ...) {
  if (object$test=="z") {
    cbind(estimate=coef(object), se=sqrt(diag(vcov(object))), df=Inf)
  } else {
    cbind(estimate=coef(object), se=sqrt(diag(vcov(object))), df=object$k-object$p)
  }
})
del2<-coef(res,select=2)
del2<-cbind(del2,(del2[,1]-del2[,5]),(del2[,1]+del2[,5]))
del2
```
